# Phytoremediation Potential of Flax Grown on Multimetal Contaminated Soils: A Field Experiment

**DOI:** 10.3390/plants13111541

**Published:** 2024-06-02

**Authors:** Danai Kotoula, Eleni G. Papazoglou, Garifalia Economou, Panayiotis Trigas, Dimitris L. Bouranis

**Affiliations:** 1 Laboratory of Systematic Botany, Department of Crop Science, Agricultural University of Athens, 11855 Athens, Greece; danai.env@gmail.com (D.K.); trigas@aua.gr (P.T.); 2 Laboratory of Agronomy, Department of Crop Science, Agricultural University of Athens, 11855 Athens, Greece; economou@aua.gr; 3 Plant Physiology and Morphology Laboratory, Department of Crop Science, Agricultural University of Athens, 11855 Athens, Greece; bouranis@aua.gr; 4 PlanTerra Institute for Plant Nutrition and Soil Quality, Agricultural University of Athens, 11855 Athens, Greece

**Keywords:** heavy metals, *Linum usitatissimum*, nitrogen fertilization, sowing period, uptake

## Abstract

The aim of this study was to assess the phytoremediation potential of fiber flax (*Linum usitatissimatum* L., var. Calista) cultivated in a soil contaminated with multiple metals, under real field conditions. A two-year (2022 and 2023) field experiment was conducted in a site contaminated with elevated concentrations of Cd, Ni, Cu, Pb, and Zn due to mining and metallurgical activities. Three different nitrogen fertilization levels were tested (N0: 0 kg N ha^−1^, N1: 30 kg N ha^−1^, N2: 60 kg N ha^−1^), and both spring and winter sowings were conducted. At full maturity, growth parameters and yields were measured. The phytoremediation potential of flax was assessed in terms of the metal concentrations in the above-ground biomass and of the metal uptake (i.e., the potential removal of the soil metals in g ha^−1^ and per year). Flax demonstrated a shorter growth cycle, with shorter and thicker plants and higher yields when sown in spring compared to winter sowing. Plant growth and productivity were not evidently influenced by additional nitrogen fertilization during plant growth. The cadmium bioaccumulation factor was 1.06, indicating that flax accumulates this metal. For Ni, Cu, Pb, and Zn, the corresponding values were 0.0, 0.04, 0.004, and 0.02, suggesting that this crop excludes these metals. The order of the higher uptake in plant tissues was as follows: Zn > Pb > Cd > Cu > Ni. In conclusion, flax demonstrated tolerance to heavy metals in the soil, effectively supporting soil restoration through cultivation. Additionally, flax showed potential as a cadmium accumulator while excluding nickel, copper, lead, and zinc.

## 1. Introduction

Soil contamination arises from the introduction of harmful substances into the soil through human activities, leading to a deterioration in soil quality. This contamination poses risks to both human health and the environment and affects the soil’s capacity to provide ecosystem services, such as the safe and adequate production of food and feed, thereby threatening global food security [1,2,3,4,5].

Europe faces widespread soil contamination, primarily located in urban areas, in mining and industrial sites and in agricultural lands. A total of 39 countries have been surveyed within the EU 27, UK, and the EIONET cooperating countries, resulting in an estimation of 2.5 million potentially contaminated sites [6,7]. Approximately 650,000 hectares of land could be classified as contaminated with organic and/or inorganic pollutants, with nearly 60% attributed to mineral oil and/or metal(loid)s [8,9]. In addition, approximately 3 billion tonnes of solid waste is discarded annually in the EU, with approx. 90 million tonnes classified as hazardous. This equates to approximately 6 tonnes of solid waste per capita [8,10].

Hence, it becomes increasingly evident that implementing remedial actions to mitigate land contamination is crucial. The use of chemical and physical methods for soil decontamination includes excavation and landfill, washing, stabilization, thermal desorption, electric field applications, etc. [11,12,13,14]. However, these methods have limitations, including high costs, inefficiency in addressing low concentrations of contaminants, and alterations to the physiochemical characteristics of the soil. In recent decades, there has been a growing interest in the development of environmentally friendly biological technologies aimed at remediating these soils at low cost [15,16,17,18]. Among them, phytoremediation uses plants to cleanse the environment by extracting, accumulating, stabilizing, and detoxifying contaminants from the substrate (soil, air, and water) through physical, chemical, or biological processes [19,20,21]. Even though phytoremediation has been scientifically proven to be effective in addressing contaminants, it still faces significant challenges, as it is time consuming and potentially harmful to living organisms due to biomagnification. Nevertheless, these drawbacks can be addressed by using non-edible industrial crops characterized by rapid growth rates and low maintenance requirements. Bast fiber crops emerge as promising candidates for phytoremediation [22,23,24].

Flax (*Linum usitatissimum* L.) is an old culture fiber crop with high potential for effective utilization in phytoremediation. It grows best in moderate climates, especially in regions receiving an annual precipitation of at least 600–650 mm, with a minimum of 110–150 mm of rain during the growing period [25]. However, there are many varieties and genotypes that are suitable for cultivation in many sites with diverse pedo-climatic conditions. An important agronomic trait of this crop is its short growing cycle, making it suitable for use in novel cropping systems, such as rotation after cereals, corn, and alfalfa.

While fiber flax serves as an important cash crop with multiple industrial applications in manufacturing biomaterials, contributing to industries such as pulp and paper, textiles, furniture, and chemicals [22,26,27,28], its cultivation area in Europe remains relatively small, approximately 140,000 hectares [29]. In recent years, there has been a growing demand for natural fibers, with flax emerging as a predominant choice. In 2012, over 51% of the total plant fiber mass used for European automotive applications was attributed to flax [30]. The European flax industry faced challenges to meet the demands of a dynamic global market that is expanding both geographically and in terms of sales outlets. The significant role that flax is anticipated to play in the European agricultural sector is underscored by the new Common Agricultural Policy (CAP). In Greece, the new CAP provides subsidies of EUR 600 per hectare to farmers cultivating flax.

Therefore, it is crucial to preserve the existing cultivated flax areas, or even expand them, and contaminated sites could potentially offer an alternative solution. The tolerance of fiber flax to toxic pollutants in soil and its capability to accumulate heavy metals have been highlighted in several studies [11,23,24,31,32,33,34]. However, most of these studies were conducted either in pots with artificially contaminated soil or through hydroponics.

The aim of this study was to assess the phytoremediation potential of flax (var. Calista) under real field conditions, when grown in soil contaminated with multiple heavy metals. The findings of this research will provide insights into the (i) potential future applications of phytoremediation using flax cultivated in contaminated areas, thereby reclaiming agricultural lands, and (ii) concentrations of heavy metals in the harvestable biomass, aiming to determine its potential uses.

## 2. Results

### 2.1. Phenological and Agronomical Traits

Flax demonstrated a shorter growth cycle, from sowing to harvest, when sown in spring compared to winter sowing, with cycle lengths of 112 days and 177 days, respectively. The growth and development of flax were significantly affected by the cultivation period (Table 1). The spring cultivation resulted in significantly shorter and thicker plants with fewer branches compared to winter cultivation.

Significant differences in height and diameter among nitrogen levels were observed only in the winter sowing of 2023 and only in N2 nitrogen levels compared to the control and N1-treated plants. However, two-way ANOVA did not reveal significant variations in the interaction between cultivation period and nitrogen level (Table 2). During spring cultivation, untreated plots showed a tendency to produce taller plants compared to treated plots, although these differences were not statistically significant, as shown in Table 1.

In contrast, during winter cultivation, plant height showed a linear increase with increasing nitrogen concentrations. An elevation in nitrogen levels was associated with an increase in the shoot diameter of flax during both cultivation periods (Table 1). At the end of the first cultivation year, there was an increasing tendency, although no significant differences were observed. However, at the end of the second cultivation year, a statistically significant difference was observed at the N2 nitrogen level. Two-way ANOVA showed significant differences in shoot diameter based on applied nitrogen levels and cultivation periods, with no significant interaction between these factors (Table 2). The evaluation of stem branching did not reveal significant differences between nitrogen levels and cultivation periods, with the cultivation period identified as the influencing factor. Non-significant differences in stem branching were observed in both spring and winter sowings, indicating similar results across cultivation periods.

### 2.2. Biomass Yield

**Figure 1:**
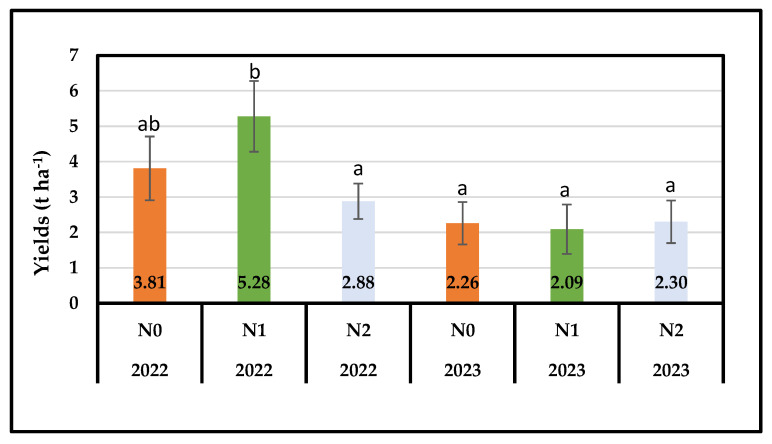
Average dry biomass yields as affected by the treatments for the two cultivation periods (±standard deviation, *p* < 0.05, ^a,b^ varying letters indicate significant differences across treatments for each cultivation period with 95% confidence level).

Flax dry biomass yields (t ha^−1^) for 2022 and 2023 are presented in Figure 1. Two-way ANOVA showed significant effects for both the cultivation period and the interaction between the cultivation period and nitrogen levels (Table 2). In spring, N1 treatment increased dry biomass compared to untreated plots but with no significant differences, while N2 treatment gave significantly lower yields compared to N1 (Figure 1). At the end of the second cultivation year, the final biomass was higher under N2 treatment, compared to N1 and the control, although no significant statistical differences were observed (Figure 1).

### 2.3. Heavy Metal Concentration and Uptake

**Figure 2:**
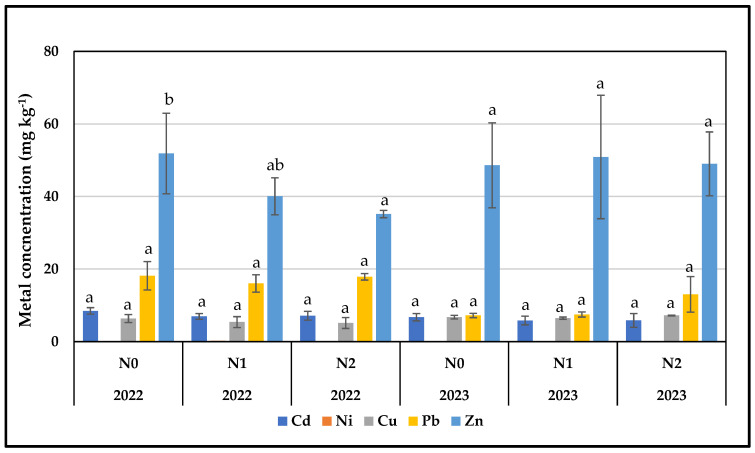
Heavy metal concentrations (mg kg^−1^) in the above-ground biomass of flax as affected by the treatments (±standard deviation, *p* < 0.05, ^a,b^ varying letters indicate significant differences across treatments for each cultivation period with 95% confidence level).

**Figure 3:**
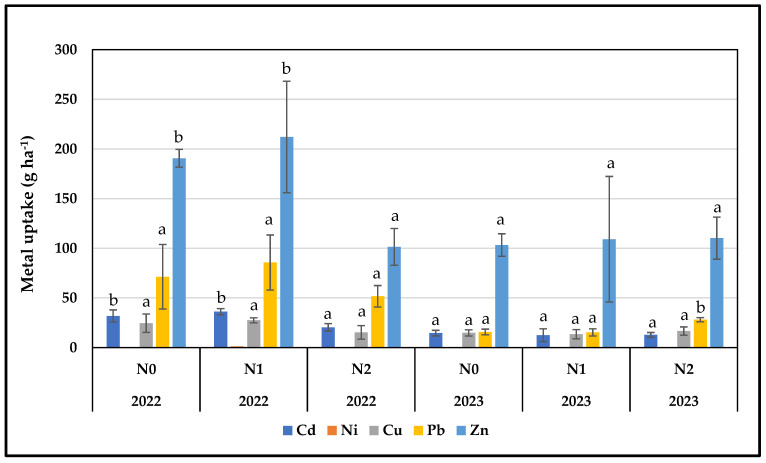
Heavy metals uptake (g ha^−1^) of flax as affected by the treatments (±standard deviation, *p* < 0.05, ^a,b^ varying letters indicate significant differences across treatments for each cultivation period with 95% confidence level).

The concentrations and uptake of Cd, Ni, Cu, Pb, and Zn in the above-ground biomass of flax for both cultivation periods and under the different nitrogen treatments are presented in Figure 2 and Figure 3. A general observation is that, although in most cases the concentrations per metal did not differ significantly between the treatments per year, the uptake was significantly higher in 2022 compared to 2023. This was probably due to the higher productivity of the crop during the first year.

The Bioconcentration Factor (BCF) shows the ability of a plant to uptake metals from the soil into its tissues. In this work, it has been calculated using the mean values for both years. This was conducted by dividing the average concentrations of each metal in the plant biomass by the average total concentrations of the corresponding metals in the soil. The results showed that the BCF for Cd was 1.06 (>1), indicating that flax tends to accumulate this metal in its above-ground biomass. Similar results for flax being able to accumulate Cd have been reported by other researchers too [32,35]. Ref. [36] investigated 166 sorghum accessions, finding that 114 had BCFs greater than 1.52 had BCFs between 0.1 and 1, and none had BCFs below 0.1. Additionally, a cadmium BCF < 1 was reported for kenaf [37], hemp [38], corn [39], miscanthus [40,41,42], and giant reed [43].

The BCF for the other metals was very low. Specifically, the BCF was 0.0 for Ni, 0.04 for Cu, 0.004 for Pb, and 0.02 for Zn. These findings indicate that, apart from Cd, which is concentrated to some extent in the plant above-ground biomass, flax does not significantly accumulate the other metals. Similar results were obtained for kenaf [44,45], ramie [46], hemp [47,48], corn [39], sunflower [49], miscanthus [50,51] and giant reed [52].

#### 2.3.1. Cd Concentration and Uptake

In 2022 and 2023, Cd concentration in the above-ground parts of flax did not show significant differences among the nitrogen treatments (N0, N1, and N2) for both cultivation periods (Figure 2). Two-way ANOVA revealed a significant difference in the cultivation period, while no significant difference was observed in the interaction between the cultivation period and applied nitrogen levels (Table 3). However, the uptake capacity of flax did not follow the same pattern as heavy metal concentration (Figure 3). In 2022, a significant difference in uptake capacity was observed among nitrogen treatments, with N0 and N1 showing higher values compared to N2, apparently due to the smaller yield of this treatment. In 2023, the untreated plots recorded the highest mean value, while no significant differences were observed among nitrogen treatments. Two-way ANOVA indicated significant differences in cultivation period, nitrogen levels, and the interaction between these factors (Table 4).

#### 2.3.2. Ni Concentration and Uptake

Nickel concentrations in flax plants for both the years 2022 and 2023 were below the detection limit, apart from N1 treatment of the first year (Figure 2). The nickel uptake capacity of flax follows the same pattern (Figure 3). Two-way ANOVA for concentration and uptake revealed significant differences in cultivation period, nitrogen levels, and the interaction between these factors (Table 3 and Table 4).

#### 2.3.3. Cu Concentration and Uptake

Copper concentrations during the spring cultivation period were decreased as the nitrogen level increased, although no significant differences were observed (Figure 2). In the winter cultivation, the Cu concentration insignificantly increased in plots treated with the N2 level of nitrogen compared to control and N1-treated plots (Figure 2). Copper accumulation (g ha^−1^) increased when flax was treated with the N1 nitrogen level in the spring sowing compared to N0 and N2 treatments, but no significant differences were determined among treatments (Figure 3). Nitrogen affected flax differently in the winter season, since increased Cu content in the harvestable parts was determined in N2-treated plots, although no significant differences were observed (Figure 3). A two-way ANOVA analysis for Cu concentration and uptake showed a significant difference between the cultivation periods, while no significant differences were observed in the interaction between the cultivation period and nitrogen level (Table 3 and Table 4).

#### 2.3.4. Pb Concentration and Uptake

In 2022, no significant differences were detected in lead concentration in flax biomass; higher but not significant concentrations were measured in the control plots, followed by the N2 and N1 treatments (Figure 2). In 2023, the lead concentration increased with the increasing applied nitrogen; the N2-treated plants stored lead in significantly higher amounts compared to N0 and N1 (Figure 2). Two-way ANOVA indicated a significant difference in the cultivation period, while no significant difference was observed in the interaction between the two tested factors—cultivation period and nitrogen level (Table 3). In 2022, the uptake capacity of Pb in flax showed an increase at the low nitrogen level compared to the control and N2-treated plants; however, no significant differences were observed among treatments (Figure 3). In the second cultivation period in 2023, significantly higher values were observed under the high nitrogen treatment (Figure 3). In the two-way ANOVA, only the cultivation period showed a significant difference, while no significant differences were observed in the interaction between the cultivation period and nitrogen level (Table 4).

#### 2.3.5. Zn Concentration and Uptake

During spring cultivation, a significant decreasing tendency in Zn concentration was noted as nitrogen-treated levels increased (Figure 2). The highest values were recorded in the control plots, followed by N1 and N2 (Figure 2). In winter flax cultivation in 2023, the concentrations of Zn did not differ significantly among the treated and control plants (Figure 2). Neither cultivation period nor nitrogen level showed a significant difference in the two-way ANOVA test, and no significant differences were observed in the interaction between factors (Table 3). In 2022, the Zn uptake capacity was significantly higher in the N0 and N1 treatment compared to the N2 treatment (Figure 3). In 2023 in terms of Zn accumulation, no significant differences were observed among treatments (Figure 3). The two-way ANOVA test showed significant differences in cultivation period and in the interaction between the two tested factors, i.e., cultivation period and nitrogen level (Table 4).

## 3. Discussion

In this study, fiber flax displayed tolerance to increased soil concentrations of heavy metals. When planted in spring as opposed to winter, a shorter growth cycle was observed, characterized by shorter and thicker plants and increased yields. Despite this, the impact of nitrogen fertilization on plant growth and productivity remained ambiguous, resulting in diverse outcomes in plant characteristics and yields across the two-year period. This inconsistency could be linked to the stress-inducing effects of heavy metals on the plants.

Previous studies underline the complex interaction of factors that impact flax development, such as residual soil nitrogen, soil type, flax cultivar, climate and moisture conditions, and growing cycle duration [33,53,54,55,56,57,58]. Moreover, the key roles of plant density, climatic conditions, and nitrogen levels in shaping stem branching in flax have been highlighted in various studies [57,58,59,60,61,62]. Understanding the importance of these factors is crucial when evaluating flax branching patterns, as they significantly affect plant architecture and yield. Nitrogen is an important factor in the growth of flax, contributing to both fiber content and stem diameter [25,53]. Its critical role points out its significance as a key nutrient for flax cultivation, affecting the quality of fiber and the structural characteristics of the plant. Moreover, water availability during growth development appears as a critical factor affecting shoot thickness [54,63]. Additionally, during the flowering and seed-filling stages, adequate water has been associated with increased biomass yields [64]. This information underlines the relationship of water supply and growth stages, influencing flax crop productivity.

The observed variations in the growth of fiber flax in our experimental field could be due to the presence of elevated concentrations of heavy metals. Heavy metals can have both direct and indirect effects on plant growth, and their impact depends on the type of metal, its concentration, and the specific plant species [37,65,66]. Ref. [67] has previously linked heavy metal exposure to reduced plant growth, suggesting a possible explanation for the observed growth variations. Heavy metals can have varying detrimental effects on plant growth by interrupting nutrient uptake, causing cellular damage, inhibiting enzymes, and interfering with various physiological processes. It is important to note that there is limited research on the direct effects of heavy metals on fiber flax in real field conditions. Commercially cultivated fiber flax typically has an average height ranging from 80 to 150 cm [25]. Studies conducted on fiber flax grown on a sand substrate containing 0.1 μM Cd showed an average height of 76 cm, compared to the control with a height of 115 cm [32]. Another pot experiment with a Cd concentration of 2 × 10^−4^ M resulted in fiber flax reaching up to 25 cm, compared with the control height of up to 50 cm [68]. Additionally, a pot study using soil artificially spiked with Cu (0, 200, 400, 600 mg kg^−1^) revealed a linear decrease in average height and dry weight (89 cm, 88 cm, 83 cm, and 60 cm, respectively) as the Cu content in the soil increased [11]. Ref. [23], in a pot experiment conducted with three different levels of Cd, Ni, Pb, and Sb in three flax cultivars, concluded that as the dose of the metal and antimony increased, the growth characteristics of the varieties decreased. The mean values of the height recorded in our study align with those reported in other field non-polluted experimental studies, resulting in similar average height values for flaxseed [57,59,69]. Ref. [37] highlighted the aspect of the growing cycle duration, reporting taller plants (62.9 cm) during autumn sowings compared to spring sowings (55.5 cm) in a semi-arid Mediterranean environment. This difference was attributed to the impact of a shorter growing cycle during spring sowings. These results are in accordance with the results of this paper. In contrast to previous investigations that established a linear correlation between nitrogen levels and height in flax [69,70,71,72], our results at the spring cultivation varied, with no significant differences between treated and control plants, while the winter cultivation results were in accordance with the findings of the other researchers.

Optimal moisture availability in the root zone results in thicker shoot diameters by increasing nutrient uptake and translocation, affecting plant growth and development [63].

Furthermore, the presence of optimal soil moisture availability is associated with the deposition of additional cellulosic layers on the primary wall, thereby promoting the development of a secondary cell wall. This process, as proposed by [54], likely contributes to a thicker shoot diameter. In contrast, low temperatures and water shortages during the winter cultivation may negatively affect shoot thickness. Elevated nitrogen levels result in more branches during both spring and winter cultivation. In spring cultivation, higher density leads to fewer branches, while winter cultivation is characterized with lower density but with a higher number of branches per plant. These findings align with [62], suggesting that flax is characterized by great phenotypic plasticity with a considerable adaptability to changes in spacing. The increased stem branching in our fiber flax cultivation agrees with [58], which noted that higher nitrogen soil concentrations were associated with an increased number of stem branches. Additionally, flax varieties considered for fiber production typically exhibit less branching, featuring thinner straw and lacking sub-stems [25]. The studied flax variety Calista, classified as a fiber flax variety, may explain its comparatively lower results compared to other studies using flaxseed.

The primary factors influencing the biomass productivity of flax include nitrogen fertilization, climatic conditions, and water availability, particularly during the flowering and seed filling stages, as well as elevated concentrations of heavy metals and metalloids [23,25,53,64,73]. Nitrogen fertilizer plays a crucial role in promoting plant growth and productivity across various crops, with studies suggesting that increased nitrogen levels lead to elevated biomass [69,70,74,75], although excessive nitrogen application may reduce total dry matter in flax [53,76]. Climatic conditions, especially wet and cold soil in spring, negatively impact flax yield by blocking plant emergence [60,64]. A water treatment of at least 120 mm during the flowering and seed filling stages in flax (May to June) results in higher yields [64]. Τhe presence of heavy metals and metalloids seems to have no visible impact on crop development and productivity when compared with other studies in non-contaminated fields. This observation aligns with the findings of [24], suggesting that in a 2-year field experiment with flax, hemp, and cotton in a mining site with increased Cd, Cu, Zn, and Pb concentrations, heavy metals did not affect the crops’ development and productivity. Our results point out that the flax variety Calista, treated with water during spring cultivation, yields increased biomass compared to winter cultivation without a water supply. The preferred nitrogen rate appears to be N1 at 30 kg N ha^−1^, resulting in higher yields in spring compared to N0 and N2, with no significant differences observed during winter.

The concentration and accumulation in the vegetative organs of flax differed between the two cultivation periods. Cadmium is a metallic element that is not essential for plant metabolism and can be toxic even at low concentrations. It tends to accumulate mainly in roots rather than shoots, with toxicity that leads to its translocation to shoots as a defense mechanism against harmful effects on roots [65,66]. In flax, studies have shown that Cd is mainly concentrated in roots, followed by shoots and, to a lower extent, seeds [24,31,77]. Reduced concentrations may result from the antagonistic effects with Pb and Zn, as both elements interact with Cd, decreasing its uptake while increasing their own intake [65]. During spring cultivation with water treatment, higher concentrations of Cd were measured, consistent with previous research [33,78]. This aligns with findings that link increased Cd concentrations in flax with greater precipitation during the growing season, indicating higher Cd movement in response to increased moisture availability. Nitrogen treatments did not seem to increase Cd concentration in flax above-ground biomass in both cultivation periods, although they did increase Cd accumulation (g ha^−1^) in the N1 treatment during spring cultivation. Our findings align with those of [79], who reported that nitrogen treatment reduced Cd content in above- and below-ground biomass of perennial and annual herbs but significantly increased Cd accumulation. The concentrations of Cd in our study were generally lower when compared to those reported by [24], except for N0 in the spring season. The observed differences could be attributed to the heterogeneity of the soil in the field, which may vary between plots and lead to variations in metal concentrations.

Nickel plays a crucial role as a micronutrient in plant biological functions, but it can become toxic at higher concentrations, even though it remains essential for plant growth at lower levels [80,81]. Nickel mobility in soil is influenced by pH, soil properties, and initial metal concentration [82]. Limited data exist on Ni accumulation in flax plants. In our field, the bioavailable concentration of Ni was low (0.8 ± 0), and Ni in above-ground flax biomass remained below the detection limit in both cultivation periods, indicating an absence of Ni accumulation. Notably, the N1 treatment was the only one that yielded detectable results in the studied flax variety. The low bioavailable Ni concentration in the soil and the fact that its origin is geogenic and not anthropogenic in the Lavreotiki area may explain the absence of Ni content in the flax tissues.

Copper plays vital roles in various physiological and biochemical reactions within plants, establishing its status as an essential nutrient. However, excessive copper levels can lead to significant toxicity, causing disruptions in bio-physiochemical processes such as growth, nutrient and water uptake, photosynthesis, root development, and leaf expansion [83]. Copper is mainly accumulated by plants through their root system, and the transfer of Cu from soil to plant varies under different soil conditions [83]. Critical soil parameters, including soil pH and organic matter, play a crucial role in controlling copper adsorption/desorption, mobility, and bioavailability in soil, influencing its uptake by plant roots [83,84]. Nitrogen application during the spring season led to a decrease in copper content, contrary to the winter season where elevated contents were observed in the highly nitrogen-treated plots. [79], suggesting that nitrogen and phosphorus fertilizers may reduce copper content in plants while increasing its uptake. The copper concentrations in our study closely align with those reported by [24]. Furthermore, climatic conditions and irrigation treatments during cultivation may also affect the copper content in flax plant tissues. These findings underscore the complex interactions between copper, soil conditions, and the agricultural practices used.

Based on the findings of [85], even at low concentrations, Pb and Cd can cause significant harm to plants, even though they are toxic and non-essential elements. In the study in [31], the bioremediation potential of flax under different concentrations of Pb, Cd, and Zn was tested. The authors found that there is a positive relationship between the increase in metal concentrations in the soil and the uptake of metals by flax plants. Our study differs from these findings, specifically for Pb, which has a higher total and bioavailable content. Despite this, the flax tissues did not show a corresponding increase in this metal, suggesting a potential antagonistic effect with Zn, known to reduce Pb uptake [65]. Lead in flax tends to be concentrated in the above-ground parts, as observed by [31], or mostly in roots with lower quantities in stems, as reported by [24]. The application of nitrogen fertilizer caused a decrease in Pb content in flax tissues in the spring period but an increase during the winter period, particularly in the high nitrogen treatment. Ref [79] observed a negative impact of nitrogen application on the concentration of Pb. However, the concentrations in our study are lower than those reported by [24]. The effect of nitrogen application on flax plants seems to be influenced by climatic conditions [53], and additionally, the plant genotype appears to be an important factor in the distribution and accumulation of heavy metals [24,31].

Zinc has a crucial role as an essential macronutrient for plant growth [86]. However, elevated concentrations of Zn can lead to toxicity [87]. Plants tend to accumulate Zn in their aerial parts, and the initial signs of toxicity are most commonly observed in the leaves [88]. Ref. [24] reported that Zn in flax, cultivated in an industrially polluted region, tends to concentrate in higher quantities in roots than in stems. In contrast, [31], in a pot experiment with varying Zn concentrations (400, 800, and 1000 mg kg^−1^ soil), found that Zn in flax mainly concentrated in above-ground parts. The use of nitrogen fertilizer did not improve the Zn content in flax tissues during spring cultivation, but a small, insignificant increase was observed, as suggested by our results during winter cultivation. The concentrations observed in our study are lower than those reported by [24]. The observed decrease in Zn content due to nitrogen application aligns with the findings of [78], which reported a lower Zn concentration in the above-ground parts of flax with nitrogen fertilization. Ref [78] further suggested that such a reduction could be attributed to the dilution of absorbed Zn due to increased biomass accumulation.

The fiber flax variety we tested is tolerant to Cd, Ni, Cu, Pb, and Zn and has the capacity to accumulate and absorb these metals in above-ground biomass in the order given: Zn > Pb > Cd > Cu > Ni, aligning with previous studies [24,31]. Climate conditions have a significant impact on the performance of the studied flax variety. Flax plants may not allow quick remediation of substantially heavy metal contaminated sites. However, the goal of flax cultivation is to gradually decrease the heavy metal content [89]. Although flax produces less biomass than some other crops, it has the advantage of using all of the harvested product, and this can be used in the textile sector, during eco-building, or to make composite furniture or automobile parts [24].

## 4. Materials and Methods

### 4.1. Site Description and Characterization

The study site is located in the Lavreotiki peninsula, an area of high geological and archaeological significance. Situated approximately 60 km southeast of Athens, Greece, it is well known for its long history and mining activities that have spanned for more than 5000 years. Mining activities in the region primarily focused on argentiferous galena, an important source of profit for ancient Aegean civilizations, likely beginning prior to 3500 BC [90]. Records indicate silver production dating back to the 7th century BC, making Lavreotiki historically important for its role in silver production, particularly for the ancient Athenian drachma. These mining activities are estimated to have yielded approximately 3500 tons of silver and around 1,400,000 tons of Pb, with a significant part (roughly 70%) extracted during the 5th and 4th centuries BC [91]. The decline of mining operations commenced in the 3rd century BC and eventually stopped altogether by the 1st century BC. Modern-era mining activities were restarted in the mid-19th century and continued until the 1980’s [92]. In 1900, the Lavrion smelters contributed 3% of the global lead production [91]. The history of mining, ore processing, and smelting has produced potentially harmful residues scattered throughout the urban and suburban areas of the modern Lavrion city [90].

The experimental field is situated northwest of the city of Lavrion, approximately 3 km away, at coordinates 37°43′59″ N, 24°02′40″ E, with an elevation of 5 m above sea level. It is characterized by mild, wet winters and hot, dry summers. Maximum temperatures, typically recorded in July and August, peak at around 31 °C, while minimum temperatures in January average around 9 °C. Annual rainfall averages are below 200 mm. Climatic data pertaining to the experimental field are provided in Appendix A.

In order to determine the soil physico-chemical properties presented in Table 5, samples were collected from two sampling points, taken from a depth of 0 to 30 cm, homogenized, air-dried, and ground to pass a 2.0 mm mesh. Soil pH was determined in a 1:1 soil/distilled water suspension after 1 h with a pH electrode. Organic matter content was measured by the Walkley–Black method [93], the cation exchange capacity (CEC) by the ammonium acetate method [94], the equivalent carbonate calcium by the Rowell method [95], and the electrical conductivity (EC) by using a conductivity meter on the soil extract obtained by shaking the soil with double-distilled water at a 1:2 (*w*/*v*) soil/water ratio. Particle size distribution was measured by the sieving and pipette method [96]. The determination of total N and available P and K was accomplished as described by [97]. The soil exhibited a low level of total nitrogen, a low level of available P, and a high level of available K and these measurements were taken into consideration for the determination of the basic fertilization of the field.

Total soil concentrations of Cd, Ni, Cu, Pb, and Zn were determined using aqua regia digestion [98]. Soil samples of 0.5 g (dry weight) were digested using a microwave oven (Model speed wave Entry DAP-60). The temperature program was as follows: 10 min at 140 °C (power 90 W), 5 min at 140 °C, and 15 min at 75 °C. The resulting solutions were cooled and then the samples were centrifuged for 10 min at 2500 rpm and were filtered through Whatman Grade 1 Qualitative Filter Paper with a pore size of 20–25 μm. Subsequently, the samples were filled to 50 mL using high-purity water. The corresponding diethylenetriaminepentaacetic acid (DTPA)-extractable fractions were measured by mixing 10 g of the soil samples with 20 mL of DTPA solution (0.005 M DTPA, adjusted to pH 7.3). The metal concentrations were quantified by an ICP-OES (PerkinElmer, Waltham, MA, USA, Model Optimal Emission Spectrometer 8000). To ensure accuracy and precision in the analyses, standard reference materials of metals (E-Merck, Darmstadt, Germany) were run with samples. The results of the total and DTPA-extractable heavy metal concentrations indicate a significant level of heavy metal contamination in the soil of the experimental field (Table 6).

### 4.2. Agronomic Practices and Experimental Set Up

In 2022, a spring sowing took place on 28 March, and the plants were harvested on July 18 of the same year. The winter sowing took place on 7 December 2022, and the plants were harvested on 1 June 2023. Cycle length was determined as the number of days from sowing to harvest and was 112 days and 177 days, respectively. During each growing season, the Flax Council of Canada guidelines were followed for tracking the development stages of plants (BBCH scale), which included emergence (BBCH 1–3), stem elongation (BBCH 4–6), flowering (BBCH 7–10), and plant maturity (harvest, BBCH 11–12) of the flax plants [99].

Before sowing, basic fertilization was applied to the field adjusted to the results of soil analyses (Table 5); more specifically, a 16-20-0 fertilizer was used in a quantity of 350 kg ha^−1^. Flax (var. Calista) seeds were sown in nine plots of 30 m^2^ each, and in a density of 100 kg ha^−1^. Three additional levels of nitrogen fertilization were tested in the form of (NH_4_)_2_SO_4_ (ammonium sulfate). These treatments were applied during the phase of stem elongation (BBCH 5) for both seasons. The applied nitrogen levels were as follows:

(i) 0 kg N ha^−1^, which referred to as N0
(ii) 30 kg N ha^−1^, which referred to as N1
(iii) 60 kg N ha^−1^, which referred to as N2

The single factor experimental design with three replications for each nitrogen treatment was the experimental design used in both seasons.

Due to late sowing in March 2022 due to low rainfall and to high temperatures, flax cultivation required supplemental water during the spring (see Appendix A). Throughout the entire cultivation period, a total of 12 irrigations were applied. Six irrigation treatments were conducted from sowing (BBCH 0) to the stage of stem elongation (BBCH 5), until late April. In early May, an additional two irrigation treatments were conducted. Two more irrigation treatments were applied at the beginning and end of June (flowering stage), with two further treatments in July as the seed-filling stage begun. The total amount of water applied was 276.0 mm, while the precipitation was 22.2 mm. The precipitation during the plant growing in the winter sowing of 2023 was 160.0 mm.

### 4.3. Plants Sampling and Measurements

When the plants reached full maturity (BBCH 12), a randomly selected 1 m^2^ area per plot was delineated. For the sampled plants, measurements were taken for height, stem diameter (using a digital caliper), number of branches per plant, and the fresh and dry weights of the above-ground plant parts. A careful washing process was carried out, which included rinsing the parts thoroughly with tap water, deionized water, and then high-purity water. The plant parts were then dried in an oven at 70 °C for 48 h, ground in a cross-hammer beater mill, and sieved through a 1 mm sieve.

The digestion of plant samples was conducted using a microwave oven (Speedwave Entry DAP-60 model) following a temperature program: 5 min at 145 °C (at 90 W power), 10 min at 190 °C (at 90 W power), and a final 10 min at 75 °C (at 90 W power). For digestion, 0.3 g of dry plant samples was digested with 5 mL of HNO_3_ and 2 mL of H_2_O_2_. The resulting solutions were cooled, brought up to a total volume of 25 mL with high-purity water, and then filtered through Whatman Grade 1 Qualitative Filter Paper with a pore size of 20–25 μm. The concentrations of Cd, Ni, Cu, Pb, and Zn were measured using an ICP-OES (Model PerkinElmer, Optimal Emission Spectrometer 8000).

### 4.4. Statistical Analysis

Statistical analysis was performed according to the experimental design using STATGRAPHICS Centurion XVII (version 1.0.1. C). As data followed a normal distribution, a one-way ANOVA was performed for comparing the growth and the phytoremediation ability of flax under the different nitrogen treatments. Furthermore, a two-way ANOVA was performed to examine if there were significant interactions between the factors of growing season and nitrogen treatment. The LSD test was used for the determination of the significant difference (*p* < 0.05) between groups at a confidence level of 95%.

## 5. Conclusions

Fiber flax is a crop with significant economic value due to its various industrial non-food applications. Utilizing it for the phytoremediation of heavy metal-contaminated sites could present an alternative solution for rehabilitating such sites across Europe. This approach could free up valuable agricultural land while providing useful biomass for further processing. The Calista variety used in this study showed a tolerance to increased soil concentrations of heavy metals. A shorter growth cycle, with shorter and thicker plants and higher yields, was observed when sown in spring compared to winter sowing. Plant growth and productivity were not evidently influenced by nitrogen fertilization, leading to diverse outcomes in plant characteristics and yields over the two years. This inconsistency could be attributed to the stress-inducing effects of heavy metals on the plants. Cadmium concentrations in the above-ground biomass were elevated, resulting in a Bioaccumulation Factor (BCF) of 1.06, indicating that flax is an accumulator of this metal. However, concentrations of other heavy metals in flax plants were minimal, with BCF values of 0.0 for Ni, 0.04 for Cu, 0.004 for Pb, and 0.02 for Zn, suggesting that this crop acts as an excluder of these metals. The uptake for all heavy metals was significantly higher in 2022 compared to 2023, likely due to the higher crop productivity in the first year. The order of the higher uptake in plant tissues was as follows: Zn > Pb > Cd > Cu > Ni. In conclusion, flax exhibited a tolerance to the presence of heavy metals in the soil, effectively supporting the soil restoration through cultivation. Additionally, flax showed potential as a cadmium accumulator while excluding nickel, copper, lead, and zinc.

## Figures and Tables

**Table 1:** Average flax height, shoot diameter, and number of branches, as affected by the treatments, for the years 2022 and 2023. (±standard deviation, *p* < 0.05, ^a,b^ varying letters indicate significant differences across treatments for each cultivation period with 95% confidence level, ^A,B^ varying letters indicate significant differences between the two cultivation periods).

Agricultural Period	Nitrogen Level	Height (cm)	Shoot Diameter (mm)	No. of Branches
Spring cultivation (2022)	N0	59.02 ± 12.0 ^a A^	3.74 ± 1.0 ^a A^	0.50 ± 0.8 ^a A^
	N1	50.73 ± 6.8 ^a A^	3.62 ± 0.9 ^a A^	0 ± 0 ^a A^
	N2	54.58 ± 6.7 ^a A^	4.53 ± 0.8 ^a A^	1.00 ± 1.3 ^a AB^
Winter cultivation (2023)	N0	76.71 ± 7.7 ^a B^	2.07 ± 0.6 ^a B^	1.50 ± 1.0 ^a B^
	N1	77.44 ± 9.1 ^a B^	2.29 ± 0.5 ^ab B^	1.61 ± 0.8 ^a B^
	N2	81.39 ± 6.2 ^b B^	2.48 ± 0.5 ^b B^	1.61 ± 0.6 ^a B^

**Table 2:** Two-way ANOVA of flax height, stem diameter, number of branches, and dry yields as affected by the cultivation period and nitrogen levels.

Factors	Height p(>F)	Stem Diameter p(>F)	No. of Tillers p(>F)	Dry Yields p(>F)
Cultivation period	0.0000	0.0000	0.0000	0.0003
Levels N	0.2592	0.0103	0.2190	0.0735
Cultivation period × Levels N	0.1544	0.2317	0.2190	0.0315

**Table 3:** Two-way ANOVA of metals concentration of flax in the above-ground biomass as affected by the cultivation period and nitrogen levels.

Factors	Cadmium p(>F)	Nickel p(>F)	Copper p(>F)	Lead p(>F)	Zinc p(>F)
Cultivation period	0.0325	0.0031	0.0249	0.0000	0.1725
Levels N	0.2049	0.0008	0.5518	0.0978	0.4242
Cultivation period × Levels N	0.9080	0.0008	0.3670	0.2062	0.3497

**Table 4:** Two-way ANOVA of metals uptake of flax as affected by cultivation period and nitrogen levels.

Factors	Cadmium p(>F)	Nickel p(>F)	Copper p(>F)	Lead p(>F)	Zinc p(>F)
Cultivation period	0.0000	0.0001	0.0151	0.0001	0.0045
Levels N	0.0207	0.0000	0.3587	0.6007	0.0594
Cultivation period × Levels N	0.0254	0.0000	0.0882	0.1138	0.0453

**Table 5:** Soil physical and chemical properties of the experimental field.

Properties	
pH	8.0–8.2 ± 0
Organic matter (%)	2.68–2.72 ± 0
CEC (%)	28.2–29.6 ± 0.99
CaCO_3_ (%)	5.04–5.46 ± 0.30
Conductivity (μS cm^−1^)	214–227 ± 9.20
Total N (g/100 g)	0.10–0.15 ± 0
Available P (mg kg^−1^)	6.6–6.9 ± 0.21
Available K (mg kg^−1^)	524–605 ± 57.30
**Mechanical Analysis**	
Clay (%)	34.0–38.0 ± 2.83
Silt (%)	45.0–49.0 ± 2.83
Sand (%)	17.0 ± 0.0
Texture	Silt-clay

**Table 6:** Total and DTPA-extractable concentrations of Cd, Ni, Cu, Pb, and Zn (mg kg^−1^) in the soil of the field at the beginning of the experiment.

Total Content (mg kg^−1^)	
Cd	6.3–6.6 ± 0.2
Ni	104.0–125.5 ± 14.8
Cu	141.0–157.1 ± 11.3
Pb	3023.5–3536.0 ± 362.7
Zn	2133.2–2343.0 ± 148.5
**Bioavailable Content (mg kg** **^−1^)**	
Cd	1.9–2.0 ± 0.1
Ni	0.8 ± 0.0
Cu	9.8–10.5 ± 0.7
Pb	471.3–580.0 ± 77.10
Zn	74.9–84.1 ± 6.4

**Figure A1:**
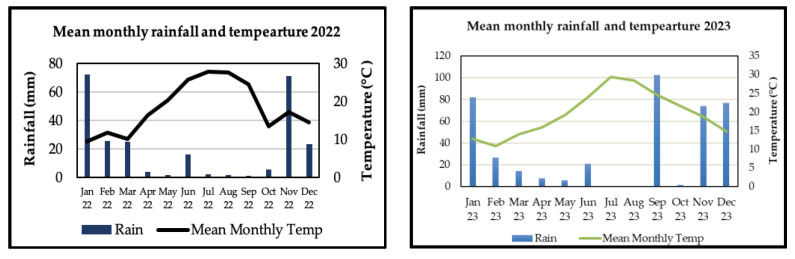
Mean monthly rainfalls and temperatures for the two experimental years (2022 and 2023) at the study site in the Lavreotiki peninsula.

## Data Availability

Data are contained within the article. The original contributions presented in the study are included in the article material; further inquiries can be directed to the corresponding authors.

## Appendix A

### Climatic Conditions

The climatic data are derived from the METEOSEARCH data base [100]. These data are recorded by a weather station which is located in the Lavreotiki peninsula. The Lavreotiki peninsula is characterized by its warm, dry summers and mild rainy winters which are typical of a Mediterranean climate. The weather conditions varied between the years 2022 and 2023 (Figure 1). In the first growing period, the spring sowing season, the highest amount of rainfall was recorded in March 2022, reaching 25.0 mm, while the lowest was in May 2022, with only 1.8 mm of precipitation. In the second growing period, the winter sowing season, the peak in rainfall was recorded in January 2023, totaling 82.2 mm, and the minimum occurred in May 2023, reaching 5.8 mm of rainfall. The growing season of 2022 was characterized by relatively warmer climatic conditions compared to the second growing period in 2023, with higher temperatures recorded from May to July 2022. In the first cultivation period, the highest average temperature reached 27.8 °C in May 2022, while the lowest was 10.1 °C in March 2022. In contrast, during the second cultivation period, mean temperatures did not exceed 24 °C, particularly in June. During most cultivated months, and more specifically from December to April, the mean temperature ranged between 10.8 °C and 15.8 °C, with May and June being the warmest months.
